# The Inflammasome Adaptor Protein ASC in Plasma as a Biomarker of Early Cognitive Changes

**DOI:** 10.3390/ijms25147758

**Published:** 2024-07-16

**Authors:** Brianna Cyr, Rosie Curiel Cid, David Loewenstein, Regina T. Vontell, W. Dalton Dietrich, Robert W. Keane, Juan Pablo de Rivero Vaccari

**Affiliations:** 1 The Miami Project to Cure Paralysis, Department of Neurological Surgery, University of Miami, Miami, FL 33136, USA; bxc205@miami.edu (B.C.); ddietrich@med.miami.edu (W.D.D.); rkeane@miami.edu (R.W.K.); 2 Center for Cognitive Neuroscience and Aging, University of Miami, Miami, FL 33136, USA; rcuriel2@med.miami.edu (R.C.C.); dloewenstein@med.miami.edu (D.L.); 3 Department of Neurology, University of Miami, Miami, FL 33136, USA; rvontell@miami.edu; 4 Department of Physiology and Biophysics, University of Miami, Miami, FL 33136, USA

**Keywords:** inflammasome, ASC, PYCARD, inflammaging, Aβ_42_, biomarker, Alzheimer’s disease, mild cognitive impairment

## Abstract

Dementia is a group of symptoms including memory loss, language difficulties, and other types of cognitive and functional impairments that affects 57 million people worldwide, with the incidence expected to double by 2040. Therefore, there is an unmet need to develop reliable biomarkers to diagnose early brain impairments so that emerging interventions can be applied before brain degeneration. Here, we performed biomarker analyses for apoptosis-associated speck-like protein containing a caspase recruitment domain (ASC), neurofilament light chain (NfL), glial fibrillary acidic protein (GFAP), and amyloid-β 42/40 (Aβ_42/40_) ratio in the plasma of older adults. Participants had blood drawn at baseline and underwent two annual clinical and cognitive evaluations. The groups tested either cognitively normal on both evaluations (N_N_), cognitively normal year 1 but cognitively impaired year 2 (N_I_), or cognitively impaired on both evaluations (I_I_). ASC was elevated in the plasma of the N_I_ group compared to the N_N_ and I_I_ groups. Additionally, Aβ_42_ was increased in the plasma in the N_I_ and I_I_ groups compared to the N_N_ group. Importantly, the area under the curve (AUC) for ASC in participants older than 70 years old in N_N_ vs. N_I_ groups was 0.81, indicating that ASC is a promising plasma biomarker for early detection of cognitive decline.

## 1. Introduction

As the elderly population increases, the prevalence of dementia has also significantly increased. The number of individuals affected by dementia is estimated to increase from 57 million to 116 million by 2040 [1]. Dementia is a term for a group of symptoms such as impairment of memory, language, problem solving, and other cognitive abilities that interfere with social and occupational function [2]. Dementia or major neurocognitive impairment is associated with many different diseases, particularly neurodegenerative diseases such as Alzheimer’s disease (AD), Lewy body dementia, and Frontotemporal dementia [3]. AD is a neurodegenerative disease that is among the top ten causes of death in the United States [2] and is characterized by progressive memory loss and cognitive decline due to the accumulation of amyloid-beta (Aβ) plaques and tau neurofibrillary tangles (NFTs) as well as neuronal death [2]. In AD, Aβ accumulation may begin to occur 25 years prior to symptoms of cognitive decline [4]. Current diagnostic methods for AD include cerebrospinal fluid (CSF) analysis for biomarkers and positron emission tomography (PET) imaging for Aβ and tau load in the brain [5,6]. These methods are invasive, costly, and usually only available at advanced medical centers. Thus, it is imperative to develop cost-effective and less-invasive early detection methods for cognitive decline. For example, blood-based biomarkers (B-BBs) have shown promise for early diagnosis of AD [7,8,9] and have been associated with Aβ and tau burden on PET and CSF biomarkers [10,11]. These brain-derived proteins can enter the peripheral circulation through transport across the blood–brain barrier as a mechanism of clearance [12]. Furthermore, during aging there are changes to the blood–brain barrier transporters that can hinder this clearance and lead to Aβ accumulation [13]. However, B-BBs are still new technologies that need to be validated for use as diagnostic tools [14].

Recent evidence has accumulated that indicates that many neurodegenerative conditions involve an inflammatory response early in the disease process [15,16,17,18]. As persons age, they typically experience a low level of chronic inflammation known as inflammaging [19]. Importantly, neuroinflammation has been linked to cognitive decline [20,21], particularly in neurodegenerative dementias associated with AD [22] and Parkinson’s disease (PD) [17]. Therefore, inflammatory proteins are promising candidates to use as biomarkers for the early diagnosis of dementia. There has been a wide variety of inflammatory proteins investigated for their biomarker potential, including cytokines, chemokines, and reactive cell markers [23,24,25,26,27]. Some of the more promising inflammatory biomarkers include glial fibrillary acidic protein (GFAP), tumor necrosis factor-α (TNFα), and MCP-1 (CCL2). Furthermore, there are mixed results regarding whether inflammatory markers are effective in predicting or monitoring disease course; however, those differences are likely due to differences in the type of fluid used, namely serum vs. plasma, as well as the sensitivity of assay used to measure the different analytes, and how patient cohorts are divided (mild cognitive impairment, AD, Aβ+/−, etc.). In addition to inflammatory markers, degenerative markers are also popular for AD B-BBs. As neurons become damaged and eventually die, they release neurofilament light chain (NfL). NfL has been found to be a reliable biomarker of neuro-axonal damage, which also increases with aging [28] and is a potential early detection biomarker for AD [27,29].

A key player that contributes to inflammaging that has not been extensively investigated as a biomarker for cognitive decline is the inflammasome, a multiprotein complex of the innate immune response which has also been associated with cognitive decline [30,31,32,33]. The inflammasome is present in many different cell types in the brain including neurons [34], astrocytes [35], and microglia [36]. The inflammasome complex becomes activated by sensing a pathogen- or damage-associated molecular pattern (PAMP or DAMP) [37]. Upon activation, the sensor protein (such as NLRP3), the adaptor protein apoptosis-associated speck-like protein containing a caspase recruitment domain (ASC), and pro-caspase-1 complex together. Then, pro-caspase-1 undergoes autocatalytic cleavage into its active form caspase-1, where it matures pro-interleukin(IL)-1β and pro-IL-18 into their active forms (IL-1β and IL-18, respectively) which propagate the inflammatory signal to surrounding cells [37]. Furthermore, excessive inflammasome activation leads to cell death, specifically pyroptosis, a process that is regulated by the cleavage of gasdermin D (GSDM-D) [38,39].

Pyroptosis leads to the secretion of the adaptor protein ASC, as well as many other components, into the extracellular space [40]. In addition to contributing to inflammaging [32], ASC oligomerizes with other proteins such as Aβ and α-synuclein in the extracellular space, hence worsening pathology in both AD and PD, respectively [41]. Additionally, ASC has been found to be elevated in the brains of AD and PD patients [42,43]. Therefore, ASC as a B-BB is suggestive of increased inflammation and cell death.

We have previously shown that inflammasome proteins are promising biomarkers of inflammation for a variety of diseases such as mild cognitive impairment [44], PD [45], multiple sclerosis (MS) [46], and traumatic brain injury [47,48]. Here, we examined the plasma concentration of ASC, NfL, GFAP, and Aβ_42/40_ as biomarkers for the early detection of cognitive decline in aged cohorts. We conducted receiver operating characteristic (ROC) curve analyses for these analytes and calculated the associated sensitivity and specificity values as well as cut-off points and negative and positive predictive values, accuracy, and likelihood ratio.

## 2. Results

### 2.1. ASC Levels Are Elevated in the Plasma of Cognitively Normal Subjects Who Went on to Develop Cognitive Decline (N_I_)

**Figure 1:**
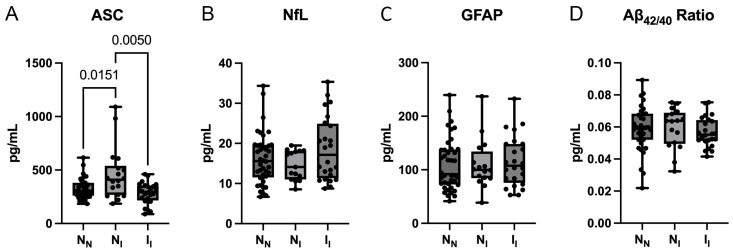
ASC plasma levels are increased in the normal–impaired (N_I_) group. Protein concentration levels in pg/mL of ASC (**A**), NfL (**B**), GFAP (**C**), and Aβ_42/40_ (**D**) in the plasma of N_N_, N_I_, and I_I_ participants. N = ASC: N_N_: 39, N_I_: 18, I_I_: 24; NfL: N_N_: 40, N_I_: 15, I_I_: 24; GFAP: N_N_: 40, N_I_: 16, I_I_: 24; Aβ_42/40_: N_N_: 35, N_I_: 17, I_I_: 24. Data presented as box plots with the minimum and maximum values showing all points. N_N_: normal–normal cognition; N_I_: normal–impaired cognition; I_I_: impaired–impaired cognition.

We have previously shown that ASC is elevated in the serum of patients with mild cognitive impairment when compared to age-matched controls and AD patients [44]. To identify potential biomarkers for the early detection of cognitive decline, we first analyzed the plasma of participants in the N_N_, N_I_, and I_I_ groups for the protein expression levels of ASC (Figure 1A), NfL (Figure 1B), GFAP (Figure 1C), and Aβ_42/40_ ratio (Figure 1D). For ASC, we found the N_N_ group to have a mean of 323.6 and a standard deviation (SD) of +/−93.27, the N_I_ group to have a mean of 460.7 (+/−243.9), and the I_I_ group to have a mean of 282.8 (+/−99.88). For NfL, we found the N_N_ group to have a mean of 16.11 (+/−6.407), the N_I_ group to have a mean of 14.59 (+/−3.664), and the I_I_ group to have a mean of 18.66 (+/−7.957). For GFAP, we found the N_N_ group to have a mean of 110.1 (+/−48.43), the N_I_ group to have a mean of 110.2 (+/−46.28), and the I_I_ group to have a mean of 115.6 (+/−47.11). For Aβ_42/40,_ we found the N_N_ group to have a mean of 0.059 (+/−0.014), the N_I_ group to have a mean of 0.06 (+/−0.013), and the I_I_ group to have a mean of 0.057 (+/−0.009). The protein levels for ASC were significantly higher in the N_I_ group compared to the N_N_ group. In addition, there was a significant decrease in the levels of ASC from the N_I_ group compared to the I_I_ group. However, there were no significant differences among the three groups for NfL, GFAP, or Aβ_42/40_. These data indicate that plasma ASC is elevated in the early stages prior to cognitive decline.

### 2.2. ASC Is a Promising Plasma Biomarker for Early Detection of Cognitive Decline

**Figure 2:**
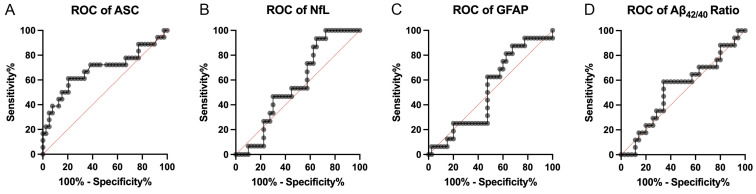
ASC in normal–normal vs. normal–impaired participants has a high area under the curve. ROC curves for ASC (**A**), NfL (**B**), GFAP (**C**), and Aβ_42/40_ (**D**) from the plasma of N_N_ and N_I_ participants. N = ASC: N_N_: 39, N_I_: 18, I_I_: 24; NfL: N_N_: 40, N_I_: 15, I_I_: 24; GFAP: N_N_: 40, N_I_: 16, I_I_: 24; Aβ_42/40_: N_N_: 35, N_I_: 17, I_I_: 24. N_N_: normal–normal cognition; N_I_: normal–impaired cognition; I_I_: impaired–impaired cognition.

To establish whether ASC could be a reliable biomarker for early detection of cognitive decline, the area under the curve (AUC) for N_N_ vs. N_I_, N_I_ vs. I_I_, and N_N_ vs. I_I_ was determined for ASC, NfL, GFAP, and Aβ_42/40_ (Table 1). Receiver operator characteristic (ROC) curves are shown for N_N_ vs. N_I_ for ASC (Figure 2A), NfL (Figure 2B), GFAP (Figure 2C), and Aβ_42_ (Figure 2D). For N_N_ vs. N_I_, ASC had the highest AUC of 0.68 (*p* = 0.03) (Table 1). The cut-off point for ASC was 329 pg/mL with 72% sensitivity and 62% specificity (Table 2). For N_I_ vs. I_I_, ASC had the highest AUC of 0.75 (*p* = 0.058) (Table 1) with a cut-off point of 326 pg/mL, 67% sensitivity and 72% specificity (Table 2). These data indicate that ASC is a promising biomarker for early detection of cognitive decline.

### 2.3. ASC, NfL, and GFAP Plasma Levels Correlate with Age

The previous literature has shown that as we age, there is an increase in levels of ASC in the brain [31,32,49]. To determine whether ASC plasma levels as well as the protein levels of NfL, GFAP, and Aβ_42/40_ are associated with aging, we ran a Spearman correlation on all participants (Table 3). We found a significant correlation between increasing levels of ASC, NfL, and GFAP with age. However, we did not see any significant changes in the levels of Aβ_42/40_, suggesting that there is a relationship between the plasma protein levels of ASC, NfL, and GFAP with aging.

### 2.4. ASC Is Elevated in the Plasma of N_I_ Participants Older Than 70 Years

**Figure 3:**
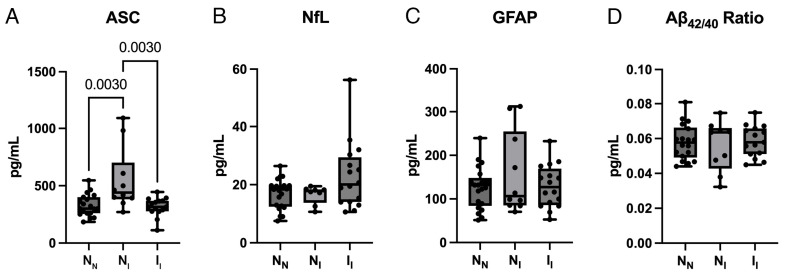
ASC plasma levels are increased in the normal–impaired group in older participants. The protein concentration levels in pg/mL of ASC (**A**), NfL (**B**), GFAP (**C**), and Aβ_42/40_ (**D**) in the plasma of N_N_, N_I_, and I_I_ participants aged 70 and older. N = ASC: N_N_: 19, N_I_: 10, I_I_: 15; NfL: N_N_: 21, N_I_: 8, I_I_: 16; GFAP: N_N_: 21, N_I_: 10, I_I_: 16; Aβ_42/40_: N_N_: 18, N_I_: 9, I_I_: 15. Data presented as box plots with the minimum and maximum values showing all points. N_N_: normal–normal cognition; N_I_: normal–impaired cognition; I_I_: impaired–impaired cognition.

**Figure 4:**
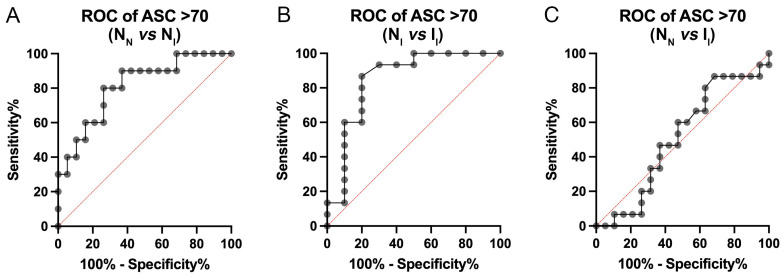
ASC has a higher AUC in older participants. ROC curves for ASC from the plasma of N_N_ vs. N_I_ (**A**), N_I_ vs. I_I_ (**B**), and N_N_ vs. I_I_ (**C**) participants. N = N_N_: 19, N_I_: 10, I_I_: 15. N_N_: normal–normal cognition; N_I_: normal–impaired cognition; I_I_: impaired–impaired cognition.

Since ASC, NfL, and GFAP correlate with age, we then analyzed the plasma levels of ASC (Figure 3A), NfL (Figure 3B), GFAP (Figure 3C), and Aβ_42/40_ (Figure 3D) among the three groups in a cohort of participants older than 70 years to determine the biomarker potential in an older cohort of individuals. For ASC, we found the N_N_ group to have a mean of 328.6 (+/−94.56), the N_I_ group 547.4 (+/−274.0), and the I_I_ group 316.7 (+/−81.42). For NfL, we found the N_N_ group to have a mean of 16.86 (+/−5.099), the N_I_ group 16.54 (+/−3.133), and the I_I_ group 22.97 (+/−11.73). For GFAP, we found the N_N_ group to have a mean of 125.6 (+/−47.73), the N_I_ group 157.8 (+/−94.41), and the I_I_ group 129.5 (+/−49.0). For Aβ_42/40_, we found the N_N_ group to have a mean of 0.058 (+/−0.01), the N_I_ group 0.056 (+/−0.014), and the I_I_ group 0.059 (+/−0.009). Our findings indicate that in this older cohort there was a significant increase in ASC from the N_N_ group to the N_I_ group, followed by a significant decrease from the N_I_ group to the I_I_ group, yet we found no significant differences among any of the groups for any of the other proteins (NfL, GFAP, and Aβ_42/40_). Therefore, to determine the potential of ASC as a reliable plasma biomarker for the early detection of cognitive decline only in those aged 70 or older, the AUC for ASC was calculated for N_N_ vs. N_I_ (Figure 4A), N_I_ vs. I_I_ (Figure 4B), and N_N_ vs. I_I_ (Figure 4C). We found that N_N_ vs. N_I_ had an AUC of 0.81 (*p* = 0.007), N_I_ vs. I_I_ had an AUC of 0.85 (*p* = 0.004), and N_N_ vs. I_I_ had an AUC of 0.51 (*p* = 0.93) (Table 4). For N_N_ vs. N_I_ the cutoff point was 386.5 pg/mL with a sensitivity of 80% and specificity of 74% (Table 5). For N_I_ vs. I_I_ the cutoff point was 389.0 pg/mL with a sensitivity of 87% and specificity of 80% (Table 5). Lastly, for N_N_ vs. I_I_, the cutoff point was 299.0 pg/mL with a sensitivity of 67% and a specificity of 42% (Table 5). Together, these results indicate that ASC is a strong early indicator for the eventual development of cognitive impairment in persons older than 70 years.

## 3. Discussion

In this study, we investigated the potential of ASC, NfL, GFAP, and Aβ_42/40_ ratio as plasma biomarkers for early detection of cognitive impairment. We analyzed three groups: those who tested cognitively normal at two visits, those who tested normal at the first visit and then impaired at the second visit, and those who tested impaired at both visits. Our results indicate that plasma levels of ASC is a promising biomarker for the early detection of cognitive decline. ASC was elevated in the plasma of N_I_ participants compared to N_N_ participants. Furthermore, ASC was significantly decreased in the plasma of the I_I_ participants compared to the N_I_ participants, suggesting that the increase in ASC may be a useful indicator during the earliest stages of cognitive decline.

The ROC analyses showed that ASC had the highest AUC values, indicating that it has a good potential for discriminative ability as a biomarker for early detection of cognitive decline. Particularly in individuals above the age of 70, there was a significant increase in the levels of ASC in the N_I_ group compared to the N_N_ with an AUC above 0.8. This finding indicates that ASC levels may be used as a biomarker to differentiate between groups.

In addition to accumulation of Aβ plaques and NFTs, inflammation is also a well-accepted event in the brain of AD patients [2]. It has been shown that Aβ plaques can induce neuroinflammation and can lead to cell death [50,51], another hallmark of AD. There is also evidence that inflammation plays an early role in the development of neurodegenerative diseases [17,52] and potentially precedes Aβ deposition [53]. More specifically, increased inflammasome activation has been linked to cognitive decline [33] and neurodegenerative diseases such as AD [42,54], MS [55,56], and PD [43,57]. However, to the best of our knowledge, there are only two published studies that have investigated whether the inflammasome protein ASC could be used as a biomarker for AD. We previously examined the biomarker potential of ASC in MCI. In that study, we found that ASC was significantly increased in the serum of MCI patients compared to controls [44]. The levels of ASC were also significantly decreased in AD patients compared to MCI patients. The other study that examined ASC as a biomarker for AD analyzed differentially expressed genes in the entorhinal cortex. ASC was found to be upregulated in two different sets of gene expression profiles for AD and ASC was found to have an AUC of 0.95 [58]. Thus, the current study is novel, since we are examining plasma instead of serum or genes and our cohort includes a group not examined before, the N_I_ group, which consists of individuals that are cognitively normal but develop cognitive impairment in the following year.

Our results support our previous findings in that levels of ASC were significantly increased in the N_I_ group compared to the N_N_ group, as well as significantly decreased in the I_I_ group compared to the N_I_ group. Mild cognitive impairment is a condition characterized by cognitive decline that is greater than expected for a person’s age and education but not severe enough to cause significant interference with activities of daily living, as occurs in more serious conditions such as dementia. Although not everyone with MCI will progress to dementia, it is considered a risk state. Amnestic MCI primarily affects memory function and conveys more dementia risk as compared to non-amnestic MCI. Recent advances in biomarker studies of persons at risk for AD and related disorders show that an individual may have abnormal biomarkers, even during preclinical/asymptomatic states. This has led to the recognition that it is important to measure cognitive impairment objectively, even prior to the MCI state. For this reason, our group and others classify persons who do not meet formal criteria for MCI, yet are not clinically/cognitively “normal.” This intermediate state between normal cognition and MCI is termed PreMCI, which is likely to be progressive over time and may represent the earliest clinically identifiable stage of AD. This study provides evidence that neuroinflammation precedes cognitive decline in elderly individuals. Furthermore, these results suggest ASC is a promising biomarker for early detection of cognitive impairment. This finding is consistent with other studies that showed inflammatory proteins such as RIG-I [59] and TREM2 [60] to be elevated early in the stages of cognitive decline and then decreased in the later stages. These findings suggest that the pattern of expression in which a protein is upregulated in the early stages of cognitive decline followed by downregulation in the later stages is perhaps characteristic of inflammation-associated proteins. Moreover, it is possible that this increased baseline level of inflammation can prime immune cells to have a more exaggerated inflammatory response when triggered. This exacerbated response can induce cognitive decline that continues to progress after the inflammatory event mediated by the innate immune response is resolved. In addition, it is likely that the decrease in ASC in blood is the result of the accumulation of ASC in the brain at the later stages of the disease. Consistent with this hypothesis, we find that ASC expression is increased in the hippocampus of donors with AD [42].

Aβ_42_ is a well-established biomarker for AD. Current methods for diagnosing AD include PET brain scanning for Aβ deposition and CSF analysis for Aβ_42_ protein levels [9,61]. However, these methods can be costly and invasive. Therefore, it is important to develop detection methods capable of diagnosing cognitive changes before the manifestation of symptoms, as pathology can begin to appear up to 25 years prior to cognitive decline [4]. Thus, early intervention is key to prevent or slow the onset of dementia, and the development of B-BB diagnostics is a cost-effective and less-invasive method for the diagnosis of AD [7,8,9]. There are mixed reports as to whether there are decreased levels of Aβ_42_ in the plasma of AD patients [29,62] or increased levels of Aβ_42_ in the plasma of AD patients [63,64,65]. Due to the inconsistent findings in Aβ_42_ as a B-BB, an increasingly popular measure is the Aβ_42/40_ ratio. Evidence shows that the levels of Aβ_42/40_ are decreased in prodromal cases of AD [66] and correspond to higher levels of cerebral Aβ deposition [67,68]. Therefore, we examined the levels of Aβ_42/40_ to determine if this measure could be used before signs of cognitive impairment. We found that there was no significant difference in the levels of Aβ_42/40_ between any of the groups, suggesting that this measure could not be used for early detection of cognitive decline. However, it is unknown whether these cognitively impaired participants will be diagnosed with AD at a later date. While this finding goes against what most of the literature shows, this could be due to the cohort of participants included in our study which were not exclusive to prodromal AD cases, and we did not correlate plasma Aβ_42/40_ with Aβ deposition in the brain.

Changes associated with inflammaging can ultimately affect plasma protein levels; therefore, it important to take age into account when determining biomarker potential for dementias. It has been previously shown that ASC, NfL, and GFAP increase with aging [28,30,69], which our results confirm. In addition, we analyzed the biomarker potential of these proteins in participants 70 years and older to determine whether age played a factor in the biomarker potential of ASC. Accordingly, ASC was the only biomarker that showed significant differences between groups, and the ROC analyses showed a better biomarker role for subjects older than 70 years than when age was not taken into consideration. These findings suggest that ASC is a strong marker of cognitive impairment, particularly in individuals above the age of 70 years old, who are at greater risk of cognitive decline. However, it is possible that this age may be lowered if cut-off points are determined with a higher sample size for younger age groups. Such a study is currently undergoing, and it may shine light onto which other inflammasome proteins can be used as biomarkers of early cognitive changes.

While this study found ASC to be a promising biomarker for the early detection of cognitive decline, it is important to note that it is unknown whether these participants had other comorbidities that were inflammatory in nature, which could contribute to the increased levels of ASC. Future studies should take into account any comorbidities that participants may have. Furthermore, it is important to note that the N_N_ and N_I_ groups skew toward heavily female (72.5% and 72% respectively). We have previously found that there is greater expression of inflammasome proteins in the cortex of female mice compared to male mice [30] and others have also found greater inflammasome protein expression in the brain of female AD mice when compared to the male brain [70]. Furthermore, it has been shown that there are sex differences in the levels of Aβ_42_ [71,72] and GFAP [73,74]. Thus, it is possible that sex could be influencing the levels of ASC between behavioral groups, as well. Age may also be an influencing factor to consider, since we examined ASC in older participants (above the age of 70), and based on the AUC, we found that ASC is an even more promising biomarker for early detection of cognitive decline when compared to the full cohort that included younger individuals.

Additionally, this study investigated biomarkers for the early detection of cognitive decline, but it is not specific for a particular disease that causes dementia. In order to determine if these biomarkers could be used for a more specific diagnosis such as AD, future studies will need to continue to monitor participants to determine if they go on to fully develop AD or another disease that can help differentiate between dementias. Current studies are underway to determine the association between these biomarkers and amyloid and tau imaging in order to confirm whether these B-BBs correlate with pathology. as well as more standard-of-care clinical chemistry testing to identify other potential biomarkers such as triglycerides and cholesterol.

## 4. Materials and Methods

### 4.1. Participants

All participants consented to be evaluated as part of an Institutional Review Board (IRB)-approved longitudinal study (IRB protocol 20140700) at the University of Miami Miller School of Medicine. All participants were recruited from the community and were required to have maintained independence to manage their daily affairs, have knowledgeable collateral informants, and did not meet DSM-5 criteria for Major Neurocognitive Disorder [75] or any other significant neuropsychiatric disorder that could preclude reliable cognitive assessments.

Each participant underwent an extensive annual evaluation. The annual visits were categorized as a baseline visit and a first annual follow-up visit. Blood was drawn during the baseline visit and plasma was collected in EDTA tubes and centrifuged at 1800× *g* for 10 min at 4 °C and stored at −80 °C until analysis. The standardized testing protocol consisted of a standard neuropsychological battery and annual clinical assessment protocol which also consisted of a clinical interview with a reliable informant using the Clinical Dementia Rating (CDR) [76] scale as well as the Mini-Mental State Examination (MMSE) [77]. Testing included, but was not limited to, the Hopkins Verbal Learning Test—Revised [78], Wechsler Memory Scale Fourth Edition Logical Memory [79], Category Fluency [80], and Trail Making Test Part A and B [81]. The neuropsychological assessments and clinical interview were completed separately by experienced clinicians to avoid criterion contamination. Participants were tested in their dominant and preferred language (English vs. Spanish).

In this study, we analyzed participants divided into 2 different groups for analysis: behavioral groups (N = 83) and behavioral groups > 70 (N = 47). The behavioral groupings were based on diagnosis following cognitive testing as stated above and were stratified into Normal–Normal (N_N_), Normal–Impaired (N_I_), and Impaired–Impaired (I_I_). The N_N_ group consisted of those who received a diagnosis of “normal cognition” at both visits. The N_I_ group consisted of those who were deemed to have “normal cognition” at their baseline visit but were deemed to have met diagnostic thresholds for clinical/cognitive impairment during an annual follow up visit. There were multiple possible diagnoses rendered if a person showed cognitive decline. These were either “Amnestic MCI” (1 individual), “Non-Amnestic MCI” (4 individuals), “PreMCI Clinical” (9 individuals), or “PreMCI Neuropsych” (4 individuals). The I_I_ group consisted of those who received a diagnosis of “Amnestic MCI” at their baseline and continued to meet diagnostic thresholds for a clinical/cognitive impairment during an annual follow up visit of either “Amnestic MCI” (18 individuals), “Non-Amnestic MCI” (5 individuals), “PreMCI Clinical” (1 individual), “PreMCI Neuropsych” (1 individual), or “dementia” (1 individual). Patient characteristics for all samples are shown in Table 6 and Table 7.

### 4.2. SimplePlex Assays

To determine the protein concentration of ASC, SimplePlex assays were performed using the ELLA system (Protein Simple) according to manufacturer’s instructions as described in [45]. Briefly, plasma samples were diluted 2×. A total of 50 µL of diluted sample was loaded into separate wells of a CART and 1 mL of washing buffer was loaded into respective wells. The assay was run with the Simple Plex Runner Software (v. 4.1.0.22) in triplicates. For ASC, the average intra-plate CV is 4.9 and the average inter-plate CV is 10.1. No data were under the lower limit of detection.

### 4.3. Single Molecule Array (Simoa) Multiplex Assays

To determine the protein concentration of NfL, GFAP, and Aβ_42/40_ the Simoa HD-X Analyzer (Quanterix) was used to run the Neurology 4-Plex E Advantage PLUST Kit according to manufacturer instructions. Briefly, activation buffer reagent was added to the RGP 2.0 Reagent bottle, and the mixture was baked at 30 °C at 800 rpm for more than 30 min and then loaded into the sample bay in the RGP rack. Calibrators and Controls were prepared and loaded into the Simoa 96-well plate together with the plasma samples. Bead reagent was resuspended and loaded into the reagent bay together with Sample Diluent, Detector Reagent, and SBG reagent in the Reagent rack. The assay was then set up in the computer and calibrators were run with the Neat protocol. Controls and samples were run with the 4× Dilution protocol and run in duplicates.

### 4.4. Biomarker and Statistical Analyses

Data obtained from the Simple and Multi-Plex assays were analyzed using Prism 10 software (v. 10.0.3) (GraphPad, San Diego, CA, USA). Outliers were removed before analysis via the ROUT method (Q = 1%) for each group and for each analysis. Receiver operating characteristics (ROCs) were calculated for ASC, NfL, GFAP, and Aβ_42/40_ ratio. The area under the curve (AUC) was determined as well as a cut-off point from a range of specificities and sensitivities, together with their negative predictive value (NPV), positive predictive value (PPV), respective likelihood ratio, and accuracy.

Following identification and removal of outliers for each group and for each analysis, data were determined to be normally distributed by the Shapiro–Wilk test. Comparison between 2 groups was carried out by an unpaired t-test for normally distributed data and by the Mann–Whitney test for data not normally distributed. Comparison between more than 2 groups was carried out by a one-way ANOVA for normally distributed data or by the Kruskal–Wallis test for data not normally distributed followed by the two-stage linear step-up procedure of Benjamini, Krieger, and Yekutieli to control for the false discovery rate. A Spearman correlation was carried out to determine how each analyte correlated with age. A *p*-value of significance was set to less than 0.05 in all tests.

## 5. Conclusions

Overall, in previous studies we have shown that ASC is a reliable biomarker of the inflammatory response in a variety of neurodegenerative diseases [45,46] and CNS injury [47,82]. Here, we highlight the role of ASC as a biomarker of the early stages of cognitive change in persons deemed at higher risk of neurodegenerative disease, which suggests that inflammation may play a critical role prior to the onset of cognitive change. Furthermore, this study provides evidence that B-BBs are a viable option for early detection of cognitive decline which will allow for a less invasive, cost-effective screening for dementia-related diseases.

## Figures and Tables

**Table 1:** ROC analysis for analyzed proteins.

Group	Biomarker	AUC	STD. Error	95% C.I.	*p*-Value
N_N_ vs. N_I_	ASC	0.684	0.085	0.517 to 0.850	0.0268
	NfL	0.57	0.078	0.418 to 0.722	0.4274
	GFAP	0.52	0.082	0.36 to 0.681	0.8136
	Aβ_42/40_	0.535	0.086	0.366 to 0.703	0.6892
N_I_ vs. I_I_	ASC	0.751	0.080	0.594 to 0.909	0.0058
	NfL	0.653	0.0877	0.481 to 0.825	0.1124
	GFAP	0.537	0.093	0.354 to 0.719	0.699
	Aβ_42/40_	0.61	0.096	0.422 to 0.798	0.2337
N_N_ vs. I_I_	ASC	0.577	0.076	0.428 to 0.726	0.3082
	NfL	0.588	0.076	0.438 to 0.737	0.2441
	GFAP	0.538	0.074	0.392 to 0.683	0.6176
	Aβ_42/40_	0.591	0.075	0.443 to 0.738	0.2409

**Table 2:** Sensitivity and specificity for analyzed proteins.

Group	Biomarker	Cut-Off Point (pg/mL)	Sensitivity (%)	Specificity (%)	PPV (%)	NPV (%)	Likelihood Ratio	Accuracy (%)
N_N_ vs. N_I_	ASC	>329.0	72	62	46	83	1.878	65
N_I_ vs. I_I_	ASC	<326.0	67	72	76	62	2.400	69

**Table 3:** Protein-level correlation with age.

Biomarker	Spearman r	*p*-Value
ASC	0.367	0.0008
NfL	0.424	<0.0001
GFAP	0.329	0.0027
Aβ_42/40_	−0.007	0.9509

**Table 4:** ROC analysis for ASC in participants >70.

Group	AUC	STD. Error	95% C.I.	*p*-Value
N_N_ vs. N_I_	0.811	0.0836	0.647 to 0.975	0.0068
N_I_ vs. I_I_	0.850	0.0909	0.672 to 1.000	0.0036
N_N_ vs. I_I_	0.509	0.102	0.310 to 0.708	0.9309

**Table 5:** Sensitivity and specificity in participants >70.

Group	Cut-Off Point (pg/mL)	Sensitivity (%)	Specificity (%)	PPV (%)	NPV (%)	Likelihood Ratio	Accuracy (%)
N_N_ vs. N_I_	>386.5	80	74	62	87	3.040	76
N_I_ vs. I_I_	<389.0	87	80	87	80	4.333	84
N_N_ vs. I_I_	>299.0	67	42	48	62	1.152	53

**Table 6:** Participant Characteristics for Behavioral Analyses.

Behavioral Group	N_N_	N_I_	I_I_
Sample Size (N)	40	18	25
Age Range	61–91	61–91	60–88
Sex			
Male	11 (27.5%)	5 (28%)	11 (44%)
Female	29 (72.5%)	13 (72%)	14 (56%)
Race			
White	35 (87.5%)	14 (78%)	21 (84%)
Black/African American	3 (7.5%)	4 (22%)	4 (16%)
Asian	2 (5%)	0 (0%)	0 (0%)
Ethnicity			
Hispanic	16 (40%)	10 (56%)	11 (44%)
Non-Hispanic	24 (60%)	8 (44%)	14 (56%)
APOE Status			
E22	0 (0%)	0 (0%)	0 (0%)
E23	6 (15%)	1 (5.5%)	4 (16%)
E24	0 (0%)	1 (5.5%)	0 (0%)
E33	19 (47.5%)	11 (61%)	11 (44%)
E34	7 (17.5%)	1 (5.5%)	5 (20%)
E44	0 (0%)	1 (5.5%)	1 (4%)
Undetermined	8 (20%)	3 (17%)	4 (16%)

**Table 7:** Participant Characteristics for >70 Analysis.

Behavioral Group	N_N_	N_I_	I_I_
Sample Size (N)	20	10	16
Age Range	70–91	71–91	70–88
Sex			
Male	4 (20%)	3 (30%)	6 (37.5%)
Female	16 (80%)	7 (70%)	10 (62.5%)
Race			
White	17 (85%)	9 (90%)	16 (100%)
Black/African American	1 (5%)	1 (10%)	0 (0%)
Asian	2 (10%)	0 (0%)	0 (0%)
Ethnicity			
Hispanic	7 (35%)	6 (60%)	8 (50%)
Non-Hispanic	13 (65%)	4 (40%)	8 (50%)
APOE Status			
E22	0 (0%)	0 (0%)	0 (0%)
E23	1 (5%)	1 (10%)	4 (25%)
E24	0 (0%)	1 (10%)	0 (0%)
E33	11 (55%)	6 (60%)	8 (50%)
E34	5 (25%)	0 (0%)	1 (6%)
E44	0 (0%)	1 (10%)	0 (0%)
Undetermined	3 (15%)	1 (10%)	3 (19%)

## Data Availability

Available data will be provided upon request to the corresponding author.
